# The Employability Process of Spanish Retired Elite Athletes: Gender and Sport Success Comparison

**DOI:** 10.3390/ijerph17155460

**Published:** 2020-07-29

**Authors:** Cristina López de Subijana, Javier Ramos, Carlos Garcia, Jose L. Chamorro

**Affiliations:** 1 Social Sciences Applied to Sport, Physical Activity and Leisure Department, Faculty of Sport Sciences-INEF, Universidad Politécnica de Madrid, 28040 Madrid, Spain; c.lopezdesubijana@upm.es (C.L.d.S.); seachel17@hotmail.com (J.R.); 2 Faculty of Sport Sciences, Universidad Europea de Madrid, Villaviciosa de Odón, 28670 Madrid, Spain; carlos.garcia@universidadeuropea.es

**Keywords:** transitions, retirement, elite athletes, employment, gender, sport success

## Abstract

The aims of the study were: (i) to describe the work integration after retirement in elite athletes, (ii) to compare the working integration of women and men, and Olympic and non-Olympic athletes, and (iii) to specify the factors that affect their employment status and current monthly income. A total of 476 former elite athletes were surveyed. Non-parametric statistics were applied to compare the differences between groups and a classification tree analysis was performed for the dependent variables. The former elite athlete’s unemployment rate was better than the general population. At the gender comparison, a wage gap appeared between women and men. At the comparison between Olympic and non-Olympic athletes, the link to first employment differed in both groups. In the prediction models, finishing higher education arose as a key factor of the working status and the monthly salary. Among those without higher education studies, planning arose as a factor determining their salary, while among those with high qualifications, gender was the key factor. This study supports the importance of a holistic view of athletic career development and it offers practical insights into the process of reaching first employment after retirement.

Since sport careers have become a subject of study, the analysis of retirement and the second career has arisen as one of the most important topics to research [1]. In this situation, incorporation into the labour market is one of the biggest challenges that athletes have to cope with. Today, the holistic model approach in career development is well recognized [2]. This model considers the athlete as a unique entity that has to face different transitions and takes into account their diverse dimensions: sport performance, development as a person, relationships with others, and the academic/vocational and financial aspects [2]. These dimensions cannot be analysed individually, but as part of a whole. The holistic approach of the model helps us to consider athletes as people that have to develop a sporting dimension, in addition to the rest of the spheres in their lives. The financial dimension explains the athletes’ evolution of economic aspects. The authors acknowledge the diversity of each individual and cultural framework of each sport. At the beginning of the sport career, athletes depend economically on the family. During their mastery stage, their incomes are mainly from sport organizations (e.g., national Olympic committees, sport federations, associations, and clubs) and/or sponsors. By the time athletes enter in the discontinuation (retirement) transition, they also slow down their engagement with sport competitions and have to deal with lower incomes, while they may have family responsibilities. After more than 20 years devoted to a sport career, elite athletes need to find a way to relocate from sport. The majority of them will need employment to continue with their life cycle. This study approaches the financial dimension of retirement, which is not commonly treated, with a quantitative methodology [3]. This research offers practical insights into the process of reaching first employment after retirement that elite sport organizations could provide to their athletes to create a smooth transition to the labour market.

## 1. Literature Review

### 1.1. Sport Retirement Transition

The figures say that about 80% of athletes experience athletic retirement as a successful transition [4,5]. This means that approximately one in every five former athletes identifies problems in their adaptation to a second career. This ratio could be higher if it is taken into account that former athletes with bad experiences after retirement presumably may be more reluctant to participate in these types of studies [6].

In a review on athletic retirement, Alfermann and Stambulova [4] identified four key factors for determining the degree of success in the adaptation: the voluntariness of the sport career termination, the degree of athletic identity, the planning of a post-sport career life and the availability of personal and social support resources during the transition. The voluntariness of the sport retirement has been widely studied [7]. Some retirements are freely chosen by the athletes (voluntary) while some others are forced by circumstances beyond the athletes’ control (involuntary). It seems that voluntary retirement increases the perceived control and is related to psychological well-being [8,9] and with a firm sense of self-efficacy [10]. On the contrary, involuntary retirements (through injury or de-selection from a team) are associated with negative emotions [11,12], a sense of betrayal and social exclusion [13,14]. The athletic identity is considered, as the athletes concentrate on a unique role involved in sport, rather than developing in other areas [15]. A strong athletic identity was identified as a barrier for planning a future beyond the sport career [16], whereas a multi-dimensional identity, in which the athlete identifies with different roles, will allow the athlete to cope successfully with the transition at the end of their sport career [17]. Planning retirement may include activities that are focused on increasing personal and social resources [18,19,20]. Being engaged in continuing studies or looking for a flexible occupation or activities related to promoting social networks in and out of the sport environment could be identified as planning [21]. Moreover, social support or social capital has been found to be an important factor to explain successful transitions to a second career. People surrounding the athlete, friends, coaches, sport and non-sport mates and especially the family influence this social capital [19,21,22,23]. Studies have found that the athletes who are engaged in career planning before their retirement have higher levels of perceived personal control. This results in them possessing higher self-efficacy in relation to their ability to successfully adapt to life after sport [23,24]. Furthermore, not planning may lead to greater difficulties in different spheres of life (family, educational and/or financial) at retirement [8,19,25].

### 1.2. Elite Athletes’ Employment

The first study on Olympic athletes’ transition to the workplace was approached by Unterlieger, with 57 Olympic athletes from USA [26]. They were all medallists, 63% had completed a college education, all of them were working and 20% had serious problems with their transitions from sport into the workplace. Another study on the financial dimension was done with a large sample of German Olympic athletes [27]. It revealed that former elite athletes had higher educational levels and achieved better employment positions than the average German population. With similar findings, a large sample of Spanish Olympic athletes revealed that their employment status and monthly income was better than the Spanish general population [6]. However, a wage penalty was evident, especially for highly qualified women.

However, reaching a satisfactory job position after an elite sport career could be seen as a matter of strategy [28]. Their study with a sample of Olympians from the Catalonia region showed how those athletes, known as the strategists, plan in advance, effectively combine their time for training and studies and preserve the social networks as a resource for the retirement process. Meanwhile, the non-strategists did not plan, did not have a higher educational level or a job that would satisfy them and would tend to choose sport as the whole lifecycle career. In the same line of research, a recent qualitative study with elite athletes from the United Kingdom explains how being involved in a training period to acquire work experience before entering the labour market or looking for a part-time job would allow them to combine their sport career with a vocational career [29]. On the other hand, the lack of flexibility on the part of the enterprises with a daily timetable or with absences due to competitions are the main barriers reflected in previous studies [30,31,32].

Moreover, results of an athletic career can also influence labour success [33]. Retired athletes who had succeeded in their sport career showed a better adjustment to post-career life [1] and had fewer occupational difficulties [33]. It appears that their sport performance facilitates the sport retirement transition [31]. Participating or not in an Olympic Games could mean a huge difference in the amount of money obtained from official scholarships, even if the athletes perform a similar training load and have the same dedication or sport achievements [18]. Moreover, “being an Olympian” has a great meaning for the athlete and the public too [34]. It is the highest level in most sport disciplines. In terms of image, employers may see that it is more beneficial to hire an Olympic athlete rather than an athlete from a marginal sport discipline [30]. For this reason, regarding the importance that the sport results may have in the entry into the labour market, elite athletes could be classified into those who have participated in the Olympic Games and those who have not.

### 1.3. Spain Framework

Spain, amongst the member states of the European Union, has the second to last position, regarding unemployment [35]. There is huge concern on the part of the national government on this topic. Thus, one theme in the agenda of each government is implementing policies for improving the unemployment rate, especially for youth. Moreover, some gender inequalities appear, named as the pay or wage gap (differences in salary). According to Eurostat [36] the wage gap in Spain is 14.9% while in the European Union, it is 16.3%. Another inequality is the reverse gender gap. According to the Organisation for Economic Co-operation and Development (OCDE), the reverse gender gap appears at some developed countries where women even with higher education than their counterparts perceive less salary per month [37]. So, there is a need to confirm the working status of the former elite athletes in Spain and if there are some differences based on gender and sport success.

This study is necessary as a quantitative research on the financial dimension with a large sample of elite athlete may contribute to exploring the process of transition into the labour market, depending on gender and sport success: particularly, participating in an edition of the Olympic Games or not. Therefore, the aims of the study were: (i) to describe the work integration after retirement in elite athletes, (ii) to compare their working integration of women and men, and in Olympic and non-Olympic athletes and (iii) to specify the factors that affect their employment status, and their current monthly income.

## 2. Methods

### 2.1. Participants

The population targeted in this study was the retired elite athletes included in the official list of the Spanish Government [38]. The sample consisted of 477 (38.9 ± 7.5 years) athletes, 298 men (62.5%) and 179 women (37.5%, see Table 1). The average time elapsed since retirement was 9 ± 6 years. Their age of retirement was 29.9 ± 6.1 years. Participants were from 32 different sports included in the Olympic sport program [39] (Athletics, Archery, Badminton, Baseball, Basketball, Boxing, Canoeing, Cycling, Fencing, Football, Gymnastics, Golf, Handball, Field Hockey, Judo, Modern Pentathlon, Horse Riding, Rowing, Rugby, Sailing, Synchronized Swimming, Shooting, Softball, Swimming, Taekwondo, Tennis, Triathlon, Volleyball, Waterpolo, Weightlifting, Winter Sports and Wrestling). A total of 308 (64.6%) of them did not participate in any edition of the Olympic Games and were classified as *non-olympics*. A total of 169 (35.4%) took part in at least one edition of Olympic Games and were classified as *olympics*. They were all Caucasian race.

### 2.2. Measures

The submitted questionnaire was based on the Social and Work Integration Questionnaire applied by Spain’s National Olympic Committee [40] and the Spanish adaptation [41] of the Athlete Retirement Questionnaire (ARQ) [4]. To assess the suitability and understanding of the questions, a pilot study was carried out with 15 athletes. The final version of the questionnaire consisted of 55 questions (54 with multiple-choice, yes/no, or ranked responses, and one open question) divided into five sections: the sociodemographic profile, sport profile, academic profile, employment, the retirement process and their current lifestyle [42].

In this article, a selection of 11 variables was taken into consideration. From the sociodemographic profile, gender (1 = Men; 2 = Women) was the only parameter considered. In the sport profile section, another variable was considered: if they participated or not in Olympic Games (1 = Yes; 2 = No). From the academic profile section, the athletes’ level of studies at their retirement (1 = No higher education; 2 = Higher education) was taken. From the employment section, three variables were selected: if they were working or not (1 = Yes; 2 = No but I am looking; 3 = No and I am not looking; those that did not have a job and were not looking for a job (*n* = 10) were not included in the analyses); how did they find their first job (i.e., 1 = Contacting the enterprise directly; 2 = Through an employment agency; 3 = Answering an add; up to 11 types), their monthly salary in one of the following ranges (1 = less than 1499 €; 2 = 1500–2499 €; 3 = over 2500 €) and if their job was related with sport (1 = Yes; 2 = No). From the retirement section, another four variables were considered: the sport retirement features as if the sport retirement process was planned, gradual and voluntary (1 = Yes; 2 = No for each feature); and if their working and economic situation was solved at retirement (1 = Yes it was completely solved; 2 = Yes It was partially solved; 3 = No I had only occasional jobs and 4 = No I had hardly anything).

### 2.3. Procedure

The retired elite Spanish athlete population was recruited using different stakeholders: the Spanish Sport Council, national sport federations, and elite athlete associations. Specifically, a snowball sampling technique was utilized [43]. The athletes in the study took part voluntarily and signed a consent form before answering the questionnaire. All the data collected were coded in order to guarantee the anonymity of the participants. The institutional ethical committee approved the study protocol (E15 11580 172).

### 2.4. Data Analysis

First of all, the main independent variables were cross checked for their independency. Gender and having participated in Olympic Games were independent (χ^2^(2) = 2.25; *p* = 0.134). Non-parametric statistics (Pearson Chi Square test) were applied to compare the differences between groups. Cramer’s V (Cv) coefficient was the effect size indicator and, in accordance with Cohen [44], considered as follows: Cv = 0.10, Cv = 0.30, and Cv = 0.50 as low, medium, and large effect sizes, respectively.

A classification tree analysis was performed for the dependent variables (the current working status and the monthly income). The algorithm used was the exhaustive CHAID (Chi-squared Automatic Interaction Detection). The Chi-Squared test identifies the relationships between independent variables through completing three steps on each node of the root (merging, splitting and stopping) to find the predictors that exert the most influence on the dependent variable [45]. The independent variables entered in the models were: gender, their participation in the Olympic games, their study level at retirement, and the features of retirement. The considerations used in the statistical analysis were: (i) significance level was set at 0.05; (ii) the maximum number of interactions was 100; (iii) the minimum change in expected cell frequencies was 0.001; (iv) the significant values adjustment was performed using the Bonferroni method; and (v) the tree had a maximum of three levels. Finally, the risk of misclassification was calculated as a measure of the reliability of the model.

## 3. Results

### 3.1. General Overview

The distribution of the employment variables is shown in Table 2. This study shows how one third (36.9%) of the former elite athletes hardly had any employment at the moment of retirement. Half of the participants (52.1%) had higher education studies. Their retirement was voluntary (80.3%), radical (67.3%) and unplanned (60.8%). They achieved their first job by means of friends or relatives (34.7%) or by sport institutions (5%). The employment was 50.5% related to sport. The unemployment rate was 10.3% and the average monthly salary was 1867 €.

### 3.2. Gender Comparison

Regarding their professional and economic situations at the moment they answered the questionnaire, whilst no significant difference appeared (*χ*^2^(3) = 7.77; *p* = 0.051; *Cv* = 0.128), women more frequently that had hardly any employment at retirement. No significant difference appeared, neither at their level of studies (*χ*^2^(1) = 0.109; *p* = 0.777), nor regarding the retirement features (*p* > 0.05 for all features). Using the working status situation, no differences were shown, (*χ*^2^(1) = 0.35; *p* = 0.553) and neither for if the work was related to sport (χ^2^(1) = 0.93; *p* = 0.34). The earning situation (monthly salary) for women was worse than for men (χ^2^(2) = 19.91; *p* < 0.001; *Cv* = 0.219).

### 3.3. Olympic and Non-Olympic Comparison

There were no significant differences between the participants at Olympic Games and those that did not participate in their professional and economic situation (*χ*^2^(3) = 1.42; *p* = 0.702), their employment situation (*χ*^2^(1) = 0.74; *p* = 0.874), monthly income (*χ*^2^(1) =0.54; *p* = 0.765) and if their job was related to sport (χ^2^(1) = 0.2.25; *p* = 0.13). No differences appeared either at their level of studies (*χ*^2^(1) = 0.39; *p* = 0.390). At the features of the retirement comparison, the Olympic athletes informed us that they planned their retirement more frequently than their counterparts (*χ*^2^(1) = 13.51; *p* < 0.001; *Cv* = 0.168). The link to their first job differed (*χ*^2^(10) = 26.16; *p* = 0.004; *Cv* = 0.250) as in the case of the Olympic athletes, it was reached more frequently through friends, relatives and sports institutions.

### 3.4. Prediction of Employment Status and Income

**Figure 1:**
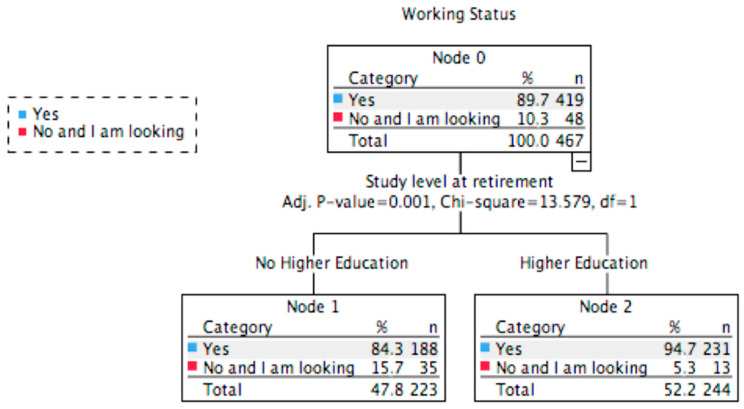
Classification tree analysis of work situation for elite athletes.

For elite athletes, the classification tree showed one factor for predicting employment status, nowadays (*χ*^2^(1) = 13.79; *p* < 0.001; Figure 1). Those that have a higher education level at retirement are predicted to have a job more easily (94.7%) than those that did not that level of studies (84.3%; Figure 1). This classification model was able to correctly classify 89.7% of the cases.

**Figure 2:**
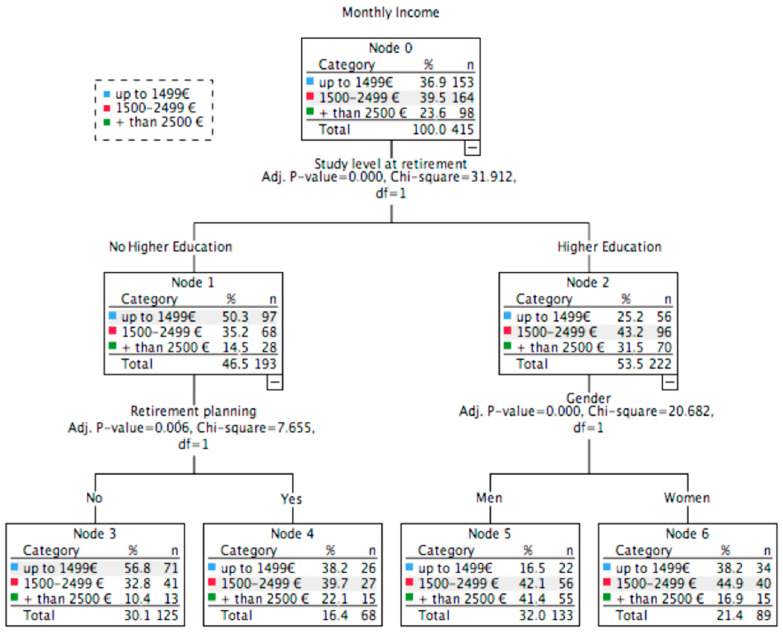
Classification tree analysis of monthly income for elite athletes.

For predicting monthly incomes in elite athletes, the classification tree showed three significant factors (*χ*^2^(1) = 31.91; *p* < 0.001; Figure 2). Again, the level of studies at retirement was the first factor. Those that have a higher education level at retirement had higher incomes per month. Then, among those with higher education studies, gender appeared as second factor. Women earned less money than men (*χ*^2^(1) = 20.68; *p* < 0.001). Furthermore, among those without higher education studies, planning is the arising factor (*χ*^2^(1) = 7.67; *p* = 0.006). Planning favourably predicts their earnings. This classification model was able to correctly classify 46.7% of the cases.

## 4. Discussion

Regarding the first aim of this article, the athletes’ work integration at retirement has been described. In spite of the fact that their average monthly income is similar to the general population’s, the athlete’s unemployment rate is better. This result is highly important taking into account that unemployment is a great concern for the Government of Spain. In relation to the second aim of the gender comparison, a wage gap appeared between women and men. At the comparison between Olympic and non-Olympic athletes, no differences were found, although the link to the first employment differed in both groups. In relation to the third aim, the key factors for reaching a job and for having a high monthly salary were determined by prediction models. It is fundamental, for both situations (working status and monthly salary), that one finishes their higher education studies. However, among those without higher education studies, planning arose as a factor determining their salary. Another factor among those with high qualifications was gender, as women’s monthly salary was lower than men’s. This study supports the importance of a holistic view of athletic career development [2] reflecting an interrelation of athletic, academic/vocational and financial dimensions.

### 4.1. Elite Athletes’ Employment Compared with the General Population

In general terms, this elite athlete sample had an unemployment rate of 10.3%, better than the 19.6% of the general population in Spain in 2016 [35]. The predicting models showed how reaching higher education studies gives them the best possibilities of acquiring a job and greater chances of having a higher monthly income. This pattern of having more chance to attain a job with a good salary with a tertiary education is the same as in the general population [46]. While the unemployment rate in the population with primary or secondary studies is 34% and 21%, respectively, the percentage in the general population with tertiary studies is 12%. Although we could only estimate the average monthly income at 1867€, it is pretty similar to the 1878€ of the general population, according to the National Statistics Institute [46]. Therefore, as previous studies remark [30] it is highly recommended for every athlete to attain a higher education level before finishing their sport career.

The next important key factor that this study shows is planning. Among those athletes without higher education, the importance of preparing this transition in advance is reflected in their monthly income. This factor has been studied several times in relation to the quality of the retirement transition [19,20,24,25,28], but never related to monthly incomes after some time. Therefore, those athletes not aiming for higher education studies should plan their retirement.

In this sense, the term dual career has been developed to refer to those athletes who combine a sports career and studies or work [47]. In the last decade, researchers [3], as well as political institutions [48], have put their efforts into the identification and promotion of social contexts and competencies that facilitate this compatibility by athletes, as well as psychological and social benefits to combine sport and studies. Carrying out a dual career also means planning a sport career and obtaining a higher level of education. Therefore, our study shows that a dual career also could have a positive impact on the employability process of retired elite athletes through monthly income.

### 4.2. Gender Differences

Although there are no significate differences between the levels of education or in sports career planning in men and women (there are works that even show that female athletes have a higher educational level and greater career planning than male athletes [49]), it is remarkable how gender differences in monthly salary appear in the elite athletes. That difference is 23% between men’s and women’s average monthly income in the whole sample. Our results are even worse than the wage gap in the Spanish general population (14.2%) provided by Eurostat [36]. The predicting model for the monthly salary showed how this gender discrimination persists within the highest qualifications group. A recent study of Olympians from Barcelona 92, Atlanta 96 and Sydney 2000 had similar results [6]. Gender differences in sport is an issue that appears throughout the entire athletic career. For example, women work more and find it more difficult to combine their sports career with their laboural life [50], women tend to earn less money in their clubs and have more difficulty finding sponsors or grants [51]. Therefore, it seems that gender discrimination appears during and after athletic career and refers more to a question of society (for example, gender stereotypes) than to a specific issue of sport. The promotion of programs that focus on gender equality in sport both during the sports career and during the employability process are especially necessary.

### 4.3. Participating or not in Olympic Games

The athletes that participate in at least one Olympic Games seem to have a privileged position in comparison with non-Olympic athletes. The fame and prestige acquired during a successful sport career could be reflected in the differences that appeared regarding the link to the first employment. Olympic athletes based this transition into the working world more on the social network from sport, including friends and relatives, than their non-Olympic counterparts. The general population reaches employment in 41.7% of the cases through relatives and friends [46], while in this sample of Olympic athletes, if we consider the sport institutions as contacts, 52% of the first employments were reached through them. Meanwhile, for the non-Olympic athletes, in only 33.1% of the cases did they reach their first job through friends, relatives or sport institutions. This result is in line with that of Vilanova and Puig [20], where the Olympians during their sport career achieved a sporting capital (recognition-popularity, social network and knowledge of the sport) that was useful at retirement. Therefore, we would presume they take advantage of this privileged situation [30].

### 4.4. Limitations

This study presents some limitations. The first one is that reaching former elite athletes is not an easy task. They do not train or congregate in a specific place. Thus, accessing them was done through different stakeholders and, to preserve their anonymity, no identity was confirmed. The second limitation was not taking into account the socioeconomic status of the family in this study, as it may constrain the future developing option of the athlete. The third one is the nature of the questions. The monthly income was presented with different salary ranges. It is difficult to answer questions relating to the personal privacy of the individual, so it was not cross-checked through any other method. The fourth limitation is in relation to the number of sport disciplines analysed. This fact may weaken our results as the sport environment varies from one sport to another [18]. The fifth limitation of this study refers to the difficult of generalization these results with any other country or situation. Still, however, this study provides valuable information from a large sample of elite athletes for stakeholders to support the elite athletes in their labour market integration.

### 4.5. Practical Implications

The practical implications of this study are related to the day by day support given to these types of athletes [52]. The first one is to continue studying until a degree is obtained, no matter how much time the athlete needs to attain it. It will be the key that will unlock the door to reaching employment. The second one is to plan the retirement transition, as this will give better chances for a better monthly salary. The third practical implication is in relation to the gender discrimination found in this study; it should warn the counsellors to monitor this situation in their athletes. Career assistance programs which promote dual careers, career planning, gender equality during the employability process and support to those athletes with less sport success are especially needed.

## 5. Conclusions

The findings of this study suggest that the process of leading former elite athletes to a professional career puts them in a better position than the general population. Still, as in the general population, the level of the studies is related with their employment status and with their monthly salary. Moreover, athletes should be proactive in terms of planning their retirement as it leads to a better job position after the sport career. Having experienced a sport career with great success, participating in the Olympic Games seems to put these athletes at the position of highest visibility in sport, so they should take advantage of that position and develop their social network. However, in the case of women, the wage penalty is still evident in the high qualifications group.

Sport stakeholders should take into account these recommendations. Professional associations and top athletes’ managers should be aware that athletes have the importance of maintaining a balanced life between the sport and social spheres. This long-term perspective will allow them to experience the retirement transition more positively. Governments, sport federations and clubs of those lesser known disciplines should provide the support and resources for athletes to combine their sport career with another vocational career (studies/work).

## Figures and Tables

**Table 1:** Sample characteristics.

		*N*	%
Gender	Men	298	62.5%
	Women	179	37.5%
Have You Ever Participate in Olympic Games?	No	308	64.6%
	Yes	169	35.4%
Study Level at Retirement	No Higher Education	228	47.9%
	Higher Education	248	52.1%
Situation at Retirement	Yes, completely solved	79	16.7%
	Yes, most of it solved	125	26.4%
	No, I had only occasional jobs	95	20.0%
	No, I had hardly anything	175	36.9%
Working Status	Yes	419	87.8%
	No, and I am looking for a job	48	10.1%
	No, and I am not looking for a job	10	2.1%

**Table 2:** Distribution of the employment process regarding on experienced the Olympic games and gender.

Situation at Retirement	Non-Olympic % (*N* = 271)	Olympic % (*N* = 144)	Men % (*N* = 296)	Women % (*N* = 178)	Total % (*N* = 474)
Yes, completely solved	18.0	14.3	17.9	14.6	16.7
Yes, most of it solved	25.5	28.0	29.4	21.3	26.4
No, I had only occasional jobs	19.3	21.4	20.3	19.7	20.0
No, I had hardly anything *	37.3	36.3	32.4	44.4	36.9
Level of studies Retirement	Non-Olympic % (*N* = 308)	Olympic % (*N* = 160)	Men % (*N* = 298)	Women % (*N* = 179)	Total % (*N* = 476)
Higher Education	53.6	49.4	51.5	53.1	52.1
No Higher Education	46.4	50.6	48.5	46.9	47.9
Retirement features	Non-Olympic % (*N* = 308)	Olympic % (*N* = 169)	Men % (*N* = 298)	Women % (*N* = 179)	Total % (*N* = 477)
Voluntary	81.2	86.3	83.0	80.5	83.0
Involuntary	18.8	13.7	17.0	19.5	17.0
Radical	64.9	71.6	67.3	69.8	67.3
Gradual	35.1	28.4	32.7	30.2	32.7
Not Planned	66.9	49.7	60.8	57.7	60.8
Planned ^†^	33.1	50.3	39.2	42.3	39.2
Link to first employment	Non-Olympic % (*N* = 272)	Olympic % (*N* = 146)	Men % (*N* = 258)	Women % (*N* = 160)	Total % (*N* = 418)
Contacting the enterprise directly	17.6	16.4	16.7	18.1	17.2
Through an employment agency	3.3	1.4	3.1	1.9	2.6
Answering an add	6.6	3.4	5.8	5.0	5.5
Applying for a public job position	10.7	7.5	8.9	10.6	9.6
Contacting friends and relatives ^†^	29.8	43.8	33.7	36.3	34.7
Specific elite athletes plan	2.6	4.1	2.3	4.4	3.1
I was already working ^†^	19.1	8.2	15.9	14.4	15.3
They contacted me	1.5	3.4	2.3	1.9	2.2
Through Sport Institutions ^†^	3.3	8.2	5.4	4.4	5.0
Self employed	4.0	3.4	5.0	1.9	3.8
After training, I was employed	1.5	0.0	0.8	1.3	1.0
Working status	Non-Olympic % (*N* = 301)	Olympic % (*N* = 166)	Men % (*N* = 293)	Women % (*N* = 174)	Total % (*N* = 467)
Yes	89.4	90.4	89.1	90.8	89.7
No and I am looking for a job	10.6	9.6	10.9	9.2	10.3
Monthly income	Non-Olympic % (*N* = 271)	Olympic % (*N* = 144)	Men % (*N* = 257)	Women % (*N* = 158)	Total % (*N* = 418)
Up to 1499 € *	37.3	36.1	30.7	46.8	36.9
1500–2499 €	40.2	38.2	38.9	40.5	39.5
More than 2500 € *	22.5	25.7	30.4	12.7	23.6
Work related with sport	Non-Olympic % (*N* = 276)	Olympic % (*N* = 146)	Men % (*N* = 258)	Women % (*N* = 158)	Total % (*N* = 416)
Yes	47.8	55.5	52.3	47.5	50.5
No	52.2	44.5	47.4	52.5	49.5

* Residuals typified over or above 1.96 for gender comparison; ^†^ residuals typified over or above 1.96 for Olympic/non-Olympic comparison.
